# Genetic Ancestry, Structural Racism, Social Determinants of Health, and Mortality Among Black Adults

**DOI:** 10.1001/jamanetworkopen.2025.10024

**Published:** 2025-05-01

**Authors:** Leonard E. Egede, Jennifer A. Campbell, Rebekah J. Walker

**Affiliations:** Division of Population Health, Department of Medicine, Jacobs School of Medicine and Biomedical Sciences, University at Buffalo, Buffalo, New York; Buffalo General Medical Center, Buffalo, New York; Division of Population Health, Department of Medicine, Jacobs School of Medicine and Biomedical Sciences, University at Buffalo, Buffalo, New York; Division of Population Health, Department of Medicine, Jacobs School of Medicine and Biomedical Sciences, University at Buffalo, Buffalo, New York

The biomedical sciences have long debated the role of race, ethnicity, and culture in population health, disease outcomes, and health disparities. Early work emphasized race as a biological basis for disease; however, research in the 1980s and 1990s identified limitations of race asa predictive biological variable in disease risk, onset, and progression.^1^ Widespread evidence now supports conceptualizing race as a social construct as opposed to a biological construct.^1,2^ Ethnicity, historically categorized as Hispanic or non-Hispanic for major US ethnic groups, and culture are also meaningful measures of social identity; however, they more appropriately capture the role a shared set of values and beliefs may have in health behavior than a biological difference across populations.^1^ More recently, genetic ancestry has emerged as a new way to understand variations in health and health disparities across populations.

Although the Human Genome Project provided evidence that humans, irrespective of race and ethnicity, are approximately 99% genetically similar, there is renewed attention on the role of genetic ancestry asa marker of disease risk and health outcomes. Genetic ancestry refers to “information about the people that an individual is biologically descended from, including their genetic relationships. Genetic information can be combined with historical information to infer where an individual’s distant ancestors lived.”^2^ While genetic ancestry provides valuable data, recognition of flaws and assumptions in using genetic ancestry as a proxy for race and ethnicity is warranted. For example, evidence shows that genetic variation and drift from generations of migration and displacement are less predictive of disease than social factors, which continue to drive outcomes.^3^ Moreover, inequity in disease risk and health outcomes are largely driven by structural factors rather than genetic factors, as shown by health disparities and racial and ethnic variations in health outcomes across leading causes of death, such as heart disease, diabetes, and hypertension.^3^

Iyer et al^4^ aimed to critically evaluate the role of genetic ancestry in explaining variation in mortality rates among a cohort of Black adults. The authors used percentage African genetic ancestry (AGA) to quantify genetic ancestry, a measure of genomic relatedness, across 21 431 single nucleotide variations (formerly, single-nucleotide polymorphisms). Within a cohort of 9685 participants in the Los Angeles County–based Multiethnic Cohort study, the authors analyzed data, including lifestyle, comorbidities, genomic analysis on blood samples, and Census tract information based on geocoded residential addresses. The cohort included adults aged 45 to 75 years who self-identified as Black, had parents who self-identified as Black, and were not foreign-born to allow examination of differences in risk patterns of Black adults. Structural racism was captured using the Index of Concentration at the Extremes based on household income and race of residents within each census tract. In addition, a composite neighborhood socioeconomic status variable was created using principal component analysis to capture median income, poverty level, median rent and home value, employment, and education within census tracts. All-cause mortality was estimated using multivariable hazard ratios, and changes in *R*^2^ for percentage AGA were assessed, with and without structural factors added to the model, using multivariable linear regression. Overall, findings showed that percentage AGA was not associated with substantial changes in the *R*^2^ compared with the association between structural factors and mortality. Findings from this study support prior evidence that structural factors, namely structural racism and structural inequalities, remain driving factors in health inequalities seen across race and ethnicity in the US.

Ancestry isan important aspect of population health, and more work is needed in this field to further understand ancestry’s contribution to health disparities across disease conditions and the underlying mechanisms at play. However, emphasis needs to remain on the structural and social determinants of health driving poor health outcomes.^5^ Continued focus on addressing upstream factors, including societal governance, macroeconomic policies, social policies, public policies, and cultural and societal values, are necessary ifwe are to see meaningful change in disparate health outcomes commonly noted in population-level health surveillance.^6,7^ In 2024, the Office of Management and Budget made significant changes to its categorization and collection of race and ethnicity data to better capture self-reported social identities tied to ancestral origin. Collection of this new measure, along with more recently developed measures of structural and social factors at a population level, is needed. More importantly, additional work is needed that collects both genetic ancestry and social measures to understand gene-environment interactions that may drive geographic and social differences in health outcomes.^5^ Instead of looking for a single measure that will explain health inequities, the scientific and medical community needs to better understand interactions across genetic, social, lifestyle, and biological factors to guide future interventional work. Specifically, work on genetic ancestry should enhance the emerging understanding of how historic and contemporary forms of structural inequalities shape health outcomes and disease risks across populations. This can be done by incorporating the study of genetic ancestry into existing frameworks for understanding the association of structural inequalities with population health. For example, the World Health Organization framework for understanding structural determinants of health inequalities incorporates factors at each level, from the socioeconomic and political context to the individual socioeconomic position to material, biological, and psychosocial factors. This framework presents existing constructs where gene-environment interactions can be examined within the context of structural inequalities.^7^ Accounting for genetic ancestry and its interaction with the surrounding societal structure and individual environment will provide meaningful data for actionable interventions that may improve overall population health and minimize health inequities.

The analysis by Iyer et al^4^ provides key information on the contribution of genetic ancestry to health inequalities. While genetic ancestry contributes to health, the contribution of structural racism is more pronounced for key outcomes, such as all-cause mortality. Evidence shows that structural racism as expressed through historical redlining, the former practice of systematically denying credit and access to financial services on the basis of race and ethnicity,^5^ is associated with lowered life expectancy and increased mortality for Black adults.^8^ Therefore, emphasis needs to be placed on understanding the role of structural factors alone and in combination with genetic ancestry, as well as the association of gene-environment interactions with health outcomes. This approach will generate actionable new knowledge that will likely lead to greater gains in life expectancy because, in the end, structural drivers of health outcomes are more mutable than ancestry or genetic factors in most cases and explain a larger proportion of variance in health outcomes.
